# ﻿Taxonomic and genetic assessment of captive White-Handed Gibbons (*Hylobateslar*) in Peninsular Malaysia with implications towards conservation translocation and reintroduction programmes

**DOI:** 10.3897/zookeys.1076.73262

**Published:** 2021-12-08

**Authors:** Millawati Gani1, Jeffrine J. Rovie-Ryan, Frankie Thomas Sitam, Noor Azleen Mohd Kulaimi, Chew Cheah Zheng, Aida Nur Atiqah, Nur Maisarah Abd Rahim, Ahmad Azhar Mohammed

**Affiliations:** 1 National Wildlife Forensic Laboratory, Ex-Situ Conservation Division, Department of Wildlife and National Parks, KM 10 Jalan Cheras, 56100 Kuala Lumpur, Malaysia Department of Wildlife and National Parks Cheras Malaysia; 2 Faculty of Resource Science and Technology, Universiti Malaysia Sarawak, 94300 Kota Samarahan, Sarawak, Malaysia Universiti Malaysia Sarawak Kota Samarahan Malaysia; 3 Faculty of Science and Technology, Universiti Kebangsaan Malaysia, 43600 UKM, Bangi Selangor, Malaysia Universiti Kebangsaan Malaysia Bangi Malaysia; 4 Faculty of Science, Universiti Putra Malaysia, 43400 UPM Serdang, Selangor, Malaysia Universiti Putra Malaysia Serdang Malaysia

**Keywords:** Control region, mitochondrial DNA, northern and southern *lar* populations, phylogenetic relationships, subspecies determination

## Abstract

Conservation translocation and reintroduction for the purpose of repopulating and reinforcing extirpated or depleted populations has been recognised as an important conservation tool, particularly for gibbon conservation in the immediate future. Feasibility assessments involving multiple factors, including taxonomic and genetic assessment of rescued and captive gibbons, are imperative prior to translocation and reintroduction programmes. In this study, we attempt to determine the subspecies and origin of captive *Hylobateslar*, White-handed gibbons, from Peninsular Malaysia to assist in future translocation and reintroduction programmes. A total of 12 captive and rescued *H.lar* samples were analysed using the control region segment of mitochondrial DNA. Sequence analyses and phylogenetic trees constructed using neighbour-joining, maximum likelihood, Bayesian inference, and network methods congruently differentiate all 12 captive individuals used in this study from other *H.lar* subspecies suggesting that these individuals belong to the *H.larlar* subspecies. In addition, two populations of *H.l.lar* were observed: (1) a southern population consisting of all 12 individuals from Peninsular Malaysia, and (2) a possible northern population represented by three individuals (from previous studies), which might have originated from the region between the Isthmus of Kra, Surat Thani-Krabi depression, and Kangar-Pattani. Our findings suggest that the complete control region segment can be used to determine the subspecies and origin of captive *H.lar*.

## ﻿Introduction

Small apes (family Hylobatidae), also known as lesser apes, consist of 20 species of gibbons inhabiting Southeast Asia which are grouped into four extant genera: *Hylobates*, *Hoolock*, *Nomascus*, and *Symphalangus*. Within the genus *Hylobates*, nine species are currently recognised (Roos 2016; IUCN 2021); *Hylobatesabbotti* Kloss, 1929, *Hylobatesagilis* F. Cuvier, 1821, *Hylobatesalbibarbis* Lyon, 1911, *Hylobatesfunereus* I. Geoffroy, 1850, *Hylobatesklossii* (Miller, 1903), *Hylobateslar* (Linneaus, 1771), *Hylobatesmoloch* (Audebert, 1798), *Hylobatesmuelleri* Martin, 1841 and *Hylobatespileatus* (Gray, 1861); two species exist in Peninsular Malaysia namely *H.agilis* and *H.lar*. In Peninsular Malaysia, *H.lar* is distributed throughout except for a narrow region between Perak River (State of Perak) and Muda River (State of Kedah) that is inhabited by the congener, *H.agilis* (Brockelman & Geissmann 2020). Both species are categorised as ‘Endangered’ by the IUCN Red List of Threatened Species (Brockelman and Geissmann 2020; Geissmann et al. 2020) and are ‘Totally Protected’ under the Wildlife Conservation Act 2010 enforced in Peninsular Malaysia. Illegal hunting for the food and pet trade as well as habitat loss due to anthropogenic activities (forest clearing for development and agriculture) have been identified as the major causes of the decline of more than 50% of *H.lar* populations in the wild across its range (Brockelman and Geissmann 2020).

Large numbers of captive gibbons kept in zoological parks (including zoos and rescue centers) are individuals rescued from the illegal pet trade, private collectors, and plantations as their habitats are cleared (Cheyne 2009; Nijman et al. 2009). Due to the threat faced by *in-situ* populations, conservation translocation and reintroduction of ex-situ populations (of rescued and captive individuals) for the purpose of repopulating and reinforcing extirpated or depleted populations has been recognised as an important conservation tool, particularly for gibbon conservation in the immediate future (Cheyne 2009; IUCN/SSC 2013; Campbell et al. 2015). However, before such conservation actions are taken, feasibility assessments involving multiple factors including taxonomic and genetic assessment of rescued and captive gibbons are imperative prior to any translocation and reintroduction programmes (Campbell et al. 2015).

To assess the taxonomic and genetic variation of gibbons, several molecular taxonomy studies have been conducted. However, most systematic studies on gibbons have focused mainly on interspecific variation (Chan et al. 2010, 2012, 2013; Israfil et al. 2011; Veeramah et al. 2015; Matsudaira and Ishida 2021) while only a few have investigated intraspecific variation (Andayani et al. 2001; Woodruff et al. 2005; Aifat and Md-Zain 2021). In particular, the taxonomic status of *H.lar* subspecies requires further examination as they are based on minor variations in body colour and fur polychromatism (Woodruff et al. 2005; Brockelman and Geissmann 2020). According to Roos et al. (2014), five subspecies of *H.lar* are currently recognised: *H.l.lar* (Linnaeus, 1771), *H.l.carpenteri* Groves, 1968, *H.l.entelloides* I. Geoffroy Saint-Hilaire, 1842, *H.l.vestitus* Miller, 1942, and *H.l.yunnanensis* Ma & Y. Wang, 1986. In this study, we employ the control region (CR) gene segment, a more variable gene segment of the mitochondrial DNA (mtDNA) (Roos and Geissmann 2001; Woodruff et al. 2005; Whittaker et al. 2007; Rovie-Ryan et al. 2014), to assess the taxonomic and genetic variation of white-handed gibbons in captivity.

## ﻿Materials and methods

### ﻿Samples and GenBank sequences

**Figure 1.**
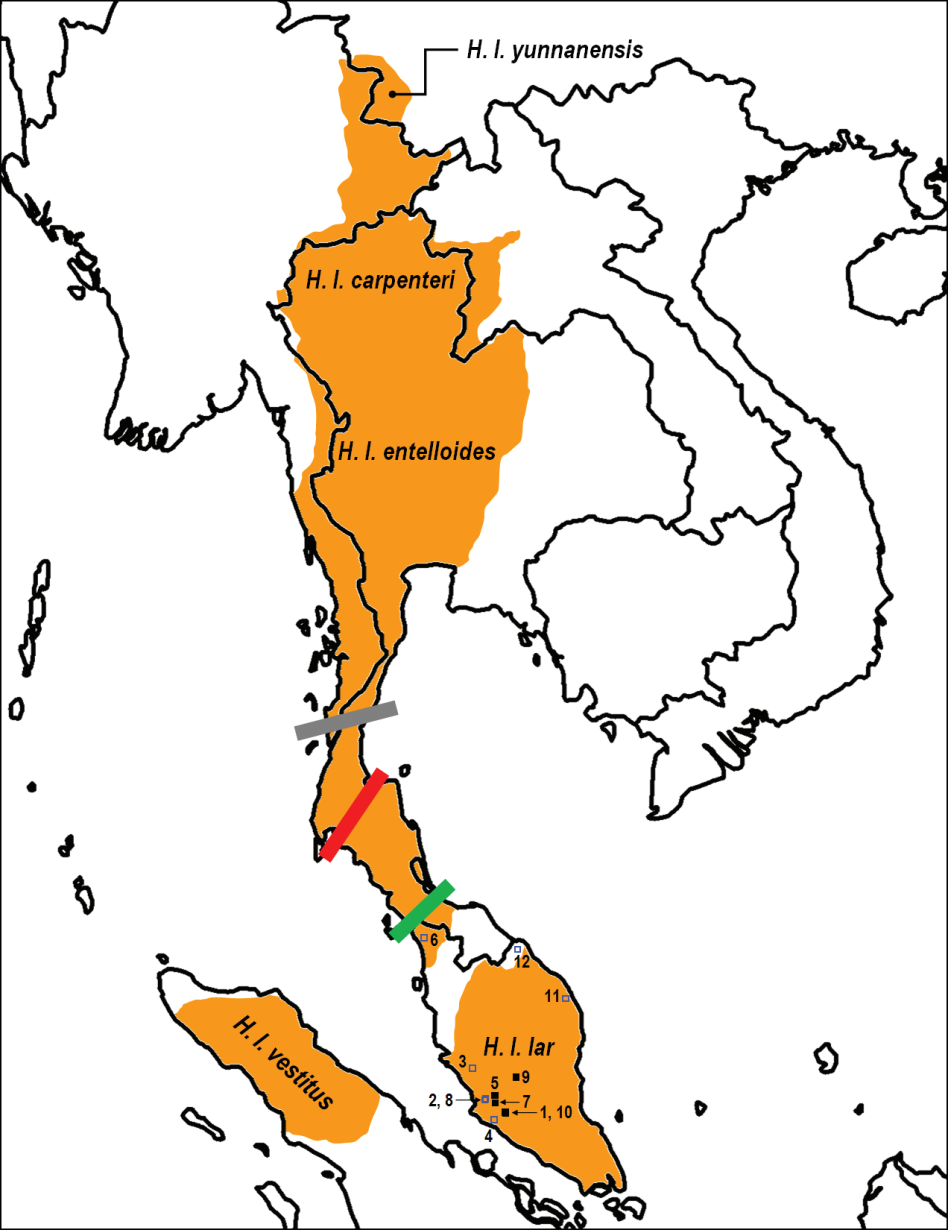
Geographical distribution of *Hylobateslar* subspecies throughout South-East Asia (adapted from Thinh et al. 2010; Brockelman and Geissmann, 2020). Black squares represent individuals of known exact locations while blue squares indicated the approximate locations of the confiscated and surrendered individuals used in this study. Numbers on the map corresponds to the location in Table 1. The approximate location of the Isthmus of Kra, the Surat Thani-Krabi depression, and the Kangar-Pattani line are indicated by the grey, red, and green lines, respectively, marking the possible break among the Indochinese (*carpenteri*, *entelloides*, and *yunnannesis*) from the *lar* subspecies.

A total of 12 unrelated *H.lar* samples were used in this study. The approximate locality of the individuals is described in Table 1 and shown in Figure 1. All rescued individuals were of known locality. The localities of the confiscated and surrendered individuals were recorded at the location of the confiscation (from dealers or private owners) or the location of the Department of Wildlife and National Parks (DWNP) offices where the animals were brought in. Currently, all of these individuals are at the National Wildlife Rescue Centre (NWRC) at Sungkai (State of Perak) and are currently under-going rehabilitation.

**Table 1.** Information of *H.lar* individuals used in this study. DWNP, Department of Wildlife and National Parks, Malaysia

No.	Sample ID	Sex	Description of locality (village, district, state)
1	Betsy	F	Rescued from Kpg. Sg. Machang, Lenggeng, Negeri Sembilan*
2	Lucy	F	Surrendered to DWNP Shah Alam, Selangor
3	Chantiq	F	Confiscated from Sungai Dusun, Selangor
4	Daly	M	Surrendered from Sepang, Selangor
5	Keramat	F	Rescued from Taman Keramat, Kuala Lumpur*
6	Abu	M	Surrendered to DWNP Alor Setar, Kedah
7	Langat	F	Rescued from Hulu Langat, Selangor*
8	Luca	M	Surrendered to DWNP Shah Alam, Selangor
9	Daru	M	Rescued from Kpg. Asli Kuala Lompat, Krau, Pahang*
10	Bella	F	Rescued from Kpg. Jeram Kedah, Lenggeng, N. Sembilan*
11.	PetPet	M	Surrendered from Kpg. Perpat, Ajil, Terengganu
12.	Lola	F	Confiscated from Pasir Mas, Kelantan

*Rescued individual were animals of known locality

Blood samples were collected during routine health checks by authorised veterinarians and personnel of DWNP where all sampling protocols adhere to the rules and regulations of the relevant authorities in Peninsular Malaysia. In addition, available mtDNACR sequences of *Hylobates* were downloaded from GenBank including the outgroup species, *Symphalangussyndactylus* (Siamang), as summarised in Suppl. material 1.

### ﻿DNA extraction, PCR amplification and DNA sequencing

Total genomic DNA was extracted from the blood samples using the QIAamp DNeasy Blood and Tissue Kit following the manufacturer’s protocol (Qiagen, Germany). We designed two new pairs of oligonucleotides to amplify the complete CR region of the mtDNA as shown in Table 2.

**Table 2.** Two pairs of newly designed oligonucleotides used in this study to amplify the complete control region gene segment of the mitochondrial DNA.

No.	Name	Oligonucleotide profile (5'–3')	Annealing temp. (°C)	Product size (bp)
1	CR1-15391F*	ACT TAA CTT CAC CCT CAG CAC C	50	550
	CR1-15887R	ACC CCC AAG TGT TGT ARG CC		
2	CR2-15810F*	YCC AGC ATC CTC CGT GAA AT	50	800
	CR2-56R*	GKG AGC CCG TCT CGA CAT TT		

*Used for DNA sequencing

PCR amplifications were conducted in 20 µl reactions using a T100 Thermal Cycler (Bio-Rad, USA) consisting of 1.0 µl of DNA template (~10 to 20 ng), 4X Green GoTaq Flexi Buffer (Promega, USA), 0.875 mM of MgCl_2_, 0.1 mM of each dNTPs, 0.1mM of each primer, 1 unit of *Taq* polymerase, and added with ddH_2_O to make up a total of 20 µl reaction mixtures. All amplifications were performed for 40 cycles each using the following profile: denaturation at 95 °C for 30 s, annealing at 50 °C for 30 s, and extension at 72 °C for 45 s, followed by a final extension step at 72 °C for 1 min. Successful PCR products were sent for sequencing on an ABI PRISM377 DNA Sequencer to a local sequencing service provider (Apical Scientific Pte. Ltd. Malaysia).

### ﻿DNA sequence analysis

DNA sequences obtained were checked for quality and aligned using the software GENEIOUS PRIME 2021.1.1 (Biomatters 2020) before being examined manually. BLAST analysis (Altschul et al. 1990) showed sequence similarities ranging from 97.2% to 98.2% (with E-values less than or equal to 0) to *H.lar* from GenBank (AF311723), providing initial confirmation to rule out the chances of amplifying the nuclear copies of mitochondrial DNA (*Numts*). Secondly, as suggested by Sorenson and Quinn (1998), we designed our oligonucleotides using the gibbon reference sequence available in GenBank to maximise the chances of amplifying the CR segment and avoiding *Numts*. Thirdly, according to Spinks and Shaffer (2007), the presence of multiple peaks in a sequenced chromatogram indicates the presence of *Numts*. We confirmed that the sequences obtained in this study showed clear single peaks indicating that our amplification has avoided amplifying *Numts*. All sequences were later registered with GenBank with the Accession Numbers MZ407482 - MZ407493.

In total, 101 CR sequences were included for subsequent analyses with aligned sequence lengths of 527-bp. DNA characteristics including conserved sites (CS), variable sites (VS), and parsimony informative sites (PIS) were checked using MEGA X (Kumar et al. 2018). Using DNASP 5.10.01 (Librado and Rozas 2009), DNA polymorphism analyses consisting of number of haplotypes (NHap), haplotype diversity (*Hd*), and nucleotide diversity, π (Nei 1987) were calculated. Genetic distances among the sequences were also calculated using the Kimura-2 parameter model (Kimura 1980) on MEGA X.

### ﻿Phylogenetic tree construction

In MEGA X, phylogenetic trees were constructed using neighbour-joining (NJ; distance based method) and maximum likelihood (ML). For the ML analysis, the HKY85 substitution model with a discrete Gamma distribution (+G) with 5 rate categories (Hasegawa et al. 1985) was determined to be the best substitution model to run the ML tree, as calculated in MEGA X. The robustness of the NJ and ML trees were assessed by bootstrapping (Felsenstein 1985) with 1000 replicates. Bayesian inference (BI) was constructed using the BEAST 2.5 package (Bouckaert et al. 2019) on two independent runs each with 10 million Markov chain Monte Carlo (MCMC) generations and sub-sampled every 1000 generations using the following settings: HKY85 substitution model with five gamma category counts, strict clock, and Yule model (Heled and Drummond 2012). The convergence of the parameters was assessed using TRACER 1.7 (Rambaut et al. 2018). The log and tree files from both runs were then combined using LOGCOMBINER before TREEANNOTATOR (both software available within the BEAST package) was then used to create a consensus tree from the combined tree files with a burn-in of 10% and a posterior probability limit of 0.5. FIGTREE 1.4.4 (Rambaut 2018) was used to visualise the BI tree. Finally, to investigate the reticulate relationship among *H.lar* haplotypes, a median-joining network (MJN) tree analysis (Bandelt et al. 1999) was performed using NETWORK 10.2 (Fluxus Technology Ltd.).

## ﻿Results

### ﻿DNA sequence characteristics, DNA polymorphism, and genetic distances

Table 3 summarised the DNA characteristics and polymorphisms of the *Hylobates* species. Sequence characteristics of *H.lar* (*N* = 57) showed 88 VS and 46 PIS. In total, 49 *lar* haplotypes were observed with π of 2.10%. Haplotype mapping revealed that the 12 individuals of *H.l.lar* from Peninsular Malaysia are unrelated with an *Hd* of 0.99.

**Table 3.** DNA characteristics and polymorphisms calculated for the *Hylobates* used in this study. *N*= number of sequences; CV= conserved sites; VS= variable sites; PIS= parsimony informative sites; NHap= number of haplotypes; *Hd*= haplotype diversity; π= nucleotide diversity.

**Species**	***N***	**DNA characteristics**			**DNA polymorphism**		
		**CV**	**VS**	**PIS**	**Nhap**	***Hd***	**π (%)**
*H.abbotti*	2	464	24	0	2	1.00	4.92
*H.agilis*	8	412	83	41	8	1.00	6.31
*H.albibarbis*	3	460	31	0	3	1.00	4.24
*H.klossii*	8	460	29	14	8	1.00	2.07
*H.lar*	57	403	88	46	49	0.99	2.10
*H.moloch*	9	454	37	15	9	1.00	2.35
*H.muelleri*	5	443	49	20	5	1.00	4.59
*H.pileatus*	8	469	23	8	8	1.00	1.51
**Total**	100	273	227	176	89	1.00	7.52

Interestingly, we observed two transversion mutations at nucleotide positions (np) 165 (thymine/cytosine to adenine) and 259 (thymine/cytosine to adenine) which differentiated all the 12 individuals used in this study from all other *H.lar* sequences (Suppl. material 2). In addition, a transition mutation at np 193 (adenine to guanine) differentiated all 12 individuals from Peninsular Malaysia, as well as three sequences from GenBank (AF311723, LC548024, and LC548028), from other sequences of *H.lar*. On the other hand, the subspecies vestitus (represented by a single sequence, LC548011) showed three transversion mutations (at np 264, 287, and 348) and 10 transition mutations (Suppl. material 2) which separated it from the other subspecies.

Pairwise genetic distances among the species of *Hylobates* are shown in Table 4. In summary, species within the genus *Hylobates* differ from each other from 7.2% (between *H.albibarbis* and *H.agilis*) to 19.93% (between *H.albibarbis* and *H.pileatus*). *Hylobateslar* differed from the other *Hylobates* species ranging from 10.46 – 14.31%. Genetic distances calculated for all *H.lar* ranged from 0.0 – 7.4% while distances among the 12 *H.lar* sequences from Peninsular Malaysia ranged from 0.2 – 3.4% (Suppl. material 3).

**Table 4.** Genetic distances (in percentage, %) calculated among the species within the genus *Hylobates* using the Kimura-2 parameter model (Kimura 1980).

No.	Species	1	2	3	4	5	6	7	8
**1**	*H.abbotti*								
**2**	*H.agilis*	13.75							
**3**	*H.albibarbis*	14.06	7.23						
**4**	*H.klossii*	13.08	10.92	12.77					
**5**	*H.lar*	11.16	12.96	13.43	12.66				
**6**	*H.moloch*	10.32	11.13	12.76	9.41	10.46			
**7**	*H.muelleri*	8.48	12.96	14.51	13.95	11.64	11.05		
**8**	*H.pileatus*	18.60	18.59	19.93	17.95	14.31	15.81	16.54	
**9**	Outgroup*	25.94	24.45	26.43	22.58	23.63	22.57	25.90	24.90

*Symphalangussyndactylus

### ﻿Phylogenetic trees and network analysis

**Figure 2.**
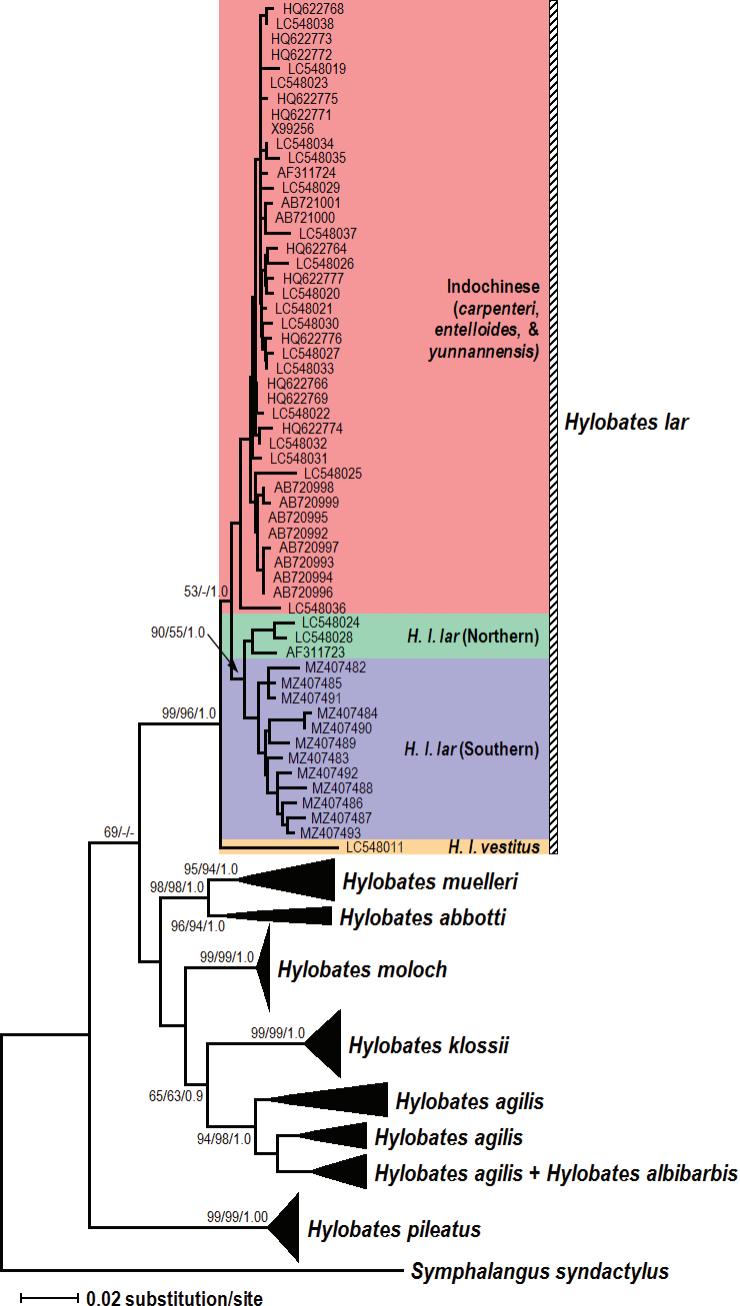
Phylogenetic relationships among the *Hylobates* species as represented by the NJ analysis. ML (Log Likelihood= -4326.23) and BI analysis produced similar topologies. Numbers above/below the branches represents bootstrap values for NJ, ML, and BI posterior probability, respectively. Only bootstrap values greater than 50% are shown.

The phylogenetic trees constructed using the NJ, ML (log likelihood= -4326.23), and BI produced similar topologies and thus was summarised using the NJ tree as shown in Figure 2. Each species formed its own monophyletic clade (with high bootstrap and posterior probability support) except for *H.albibarbis*, which clustered within the *H.agilis* clade. *Hylobatespileatus* was the basal species of the genus *Hylobates* although with low support (below 50% support). *Hylobateslar* was separated from all the other species with low to moderate support. *Hylobatesabbotti* and *H.muelleri* clustered together to form the Bornean species group (except for *H.albibarbis*) while *H.moloch*, *H.klossii* and *H.agilis* formed the Indonesian species group.

Within *H.lar*, we observed three possible subspecies groupings: (1) the basal *H.l.vestitus*, (2) *H.l.lar* (consisting of all 12 captive individuals from Peninsular Malaysia as well as the three sequences from GenBank mentioned above), and (3) a possible Indochinese subspecies group (representing *H.l.entelloides*, *H.l.carpenteri*, and *H.l.yunnanensis*). The Indochinese subspecies group did not show any obvious groupings according to subspecies. The *H.l.lar* group further splits with strong support (bootstrap and posterior probability) into two subgroups, which we tentatively define as representing northern and southern populations. The presumed northern *H.l.lar* population consists of three sequences from GenBank (of unknown origins) while the southern population consists of all 12 captive individuals from Peninsular Malaysia.

**Figure 3.**
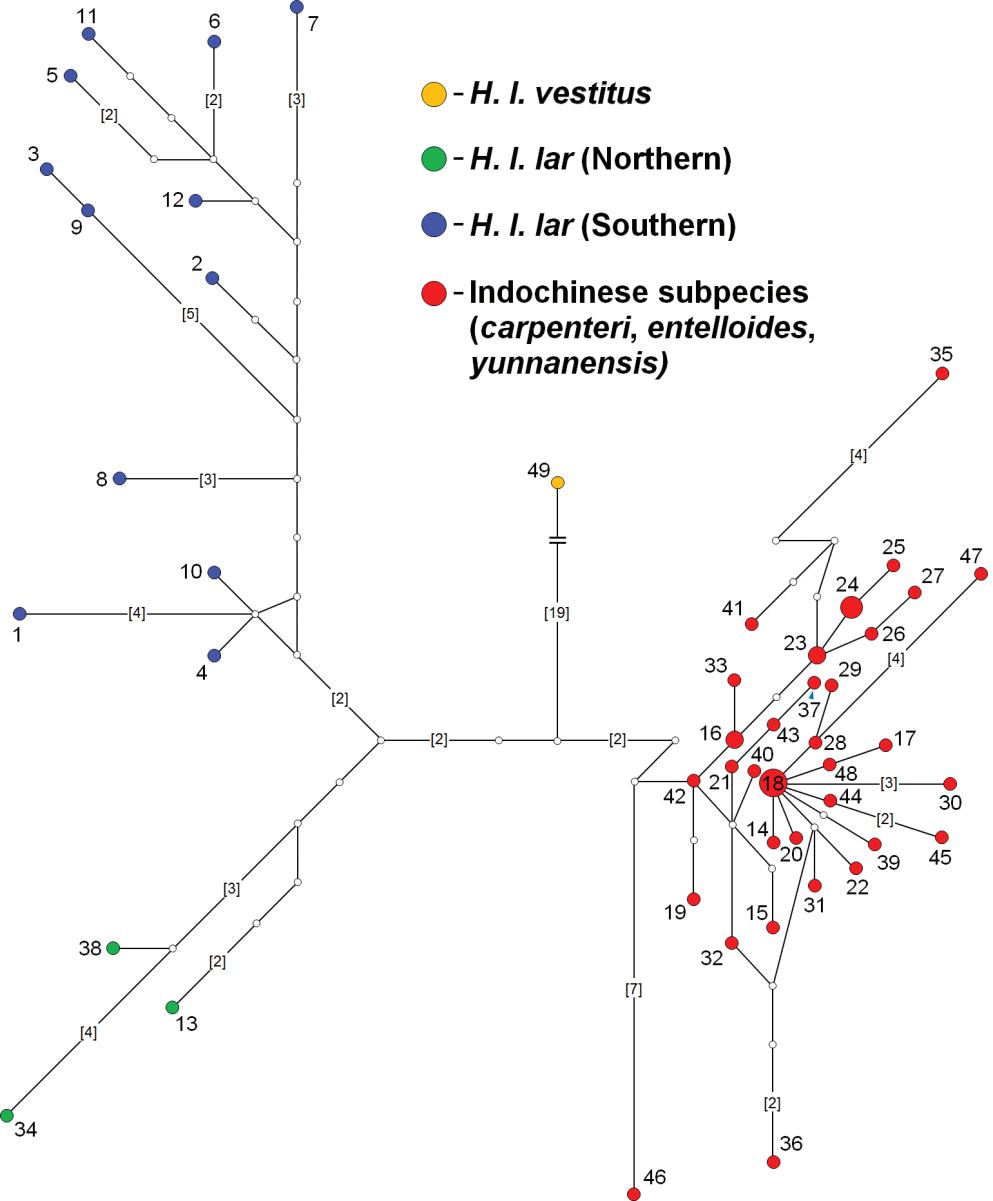
Median-joining network (MJN) constructed showing the relationships among the *H.lar* haplotypes. Each circle size is proportional to the number of individuals in each haplotype. The numbers next to the nodes correspond to the haplotype designation as listed in Supplementary Material, Table S1. The lines connecting the haplotypes represent single mutations unless indicated otherwise (numbers in parentheses). Hypothetical haplotypes (median vectors) are represented by white circles.

Similarly, the MJN tree constructed using the *H.lar* haplotypes (*N* = 49) produced similar groupings as the phylogenetic trees (Fig. 3). *Hylobatesl.vestitus* differs from the Indochinese subspecies haplotypes and *H.l.lar* haplotypes (northern and southern) by at least 23 and 26 mutational steps, respectively. The northern *H.l.lar* haplotypes differ from the southern *H.l.lar* haplotypes by at least 10 mutational steps.

## ﻿Discussion

A total of 1030-bp of the complete CR of mtDNA was successfully obtained from all 12 samples used in this study using the newly designed pairs of oligonucleotides. The CR segment of mtDNA is the most variable region and has been recommended as the appropriate segment to be used to infer gibbon phylogeny (Roos and Geissmann 2001; Woodruff et al. 2005; Whittaker et al. 2007; Matsudaira and Ishida 2010; Aifat and Md-Zain 2021). However, due to the lack of complete CR sequences of *Hylobates* in GenBank, a total of 527-bp of aligned dataset were analysed from 100 sequences representing all species of *Hylobates* except *H.funereus*. Our initial analysis of the complete CR sequence of *H.lar* (aligned sequence length of 1110-bp) showed an additional 59 VS and 34 PIS which may provide more informative data for the taxonomic and genetic assessment of *H.lar*. Therefore, we recommend the continued use of the complete CR segment in future studies of gibbon phylogeny and taxonomy.

The phylogenetic relationships among *Hylobates* remain unresolved. Firstly, we observed *H.pileatus* as the basal species of *Hylobates* (although with low support), a result that is consistent with previous studies (Israfil et al. 2011; Chan et al. 2013; Matsudaira and Ishida 2021). Secondly, the relationships among the Sundaic species group (*abbotti*, *agilis*, *albibarbis*, *klossii*, *moloch*, and *muelleri*) remains conflated particularly with regard to the basal species of this group (Thinh et al. 2010; Chan et al. 2013; Matsudaira and Ishida 2021). In this study, the Bornean species group (*abbotti* and *muelleri*) diverged first followed by the Indonesian species group (*agilis*, *klossii*, and *moloch*). The clustering of *H.albibarbis* within the H.agilis clade is expected because the subspecies albibarbis was initially classified as a subspecies of *H.agilis* (Thinh et al. 2010; Matsudaira and Ishida 2021).

The phylogenetic positioning of *H.lar* within the genus *Hylobates* concurs with findings from previous studies (Israfil et al. 2011; Chan et al. 2013; Matsudaira and Ishida 2021). Our phylogenetic and network analysis showed that *H.l.vestitus* from Sumatra diverged first (Thinh et al. 2010; Aifat and Md-Zain 2021; Matsudaira and Ishida 2021) before the proto-*H.lar* bifurcated to form the *H.l.lar* cHylobateslade and the possible Indochinese subspecies clade (*entelloides*, *carpenteri*, and *yunnanensis*). However, the Indochinese subspecies clade did not show any obvious subspecies groupings. Note that most of the sequences of the Indochinese subspecies clade were obtained from zoo individuals of unknown origin and locality (Suppl. material 1) except for sequences by Matsudaira et al. (2013) (AB720992 - AB721001) which originated from individuals from Khao Yai National Park, Thailand representing the subspecies *H.l.entelloides*. Thus, we deduce these following scenarios to explain the condition: (1) Indochinese *lar* gibbons are represented by only one subspecies (*entelloides*) instead of three, (2) sequences of *lar* gibbons from GenBank of unknown locality were derived from only one subspecies, *H.l.entelloides*, and (3) the CR segment is not powerful enough to differentiate among the Indochinese subspecies. In view of the lack of information regarding the origin of the zoo animals as well as the lack of reference samples of known locality, these proposed scenarios must be regarded as hypothetical and preliminary. Future studies should therefore use individuals of known localities to correctly assign captive and rescued gibbons to their geographical provenance and origin.

Finally, our findings revealed that all 12 captive individuals used in this study belong to the *H.l.lar* subspecies. Aifat and Md-Zain (2021) used partial cytochrome *b* gene segment of the mtDNA to successfully identify the subspecies of captive *H.lar* in Peninsular Malaysia but suggested that the CR segment be used in further studies on *H.lar*. The CR segment has been used in forensic investigations of various wildlife species to identify the subspecies and geographical origins of rescued or confiscated animals (Iyengar 2014). Interestingly, our results suggest the occurrence of two distinct populations of *H.l.lar*, as demarcated by two transversion mutations at np 165 and 259: (1) a southern population consisting of all 12 individuals from Peninsular Malaysia, and (2) a possible northern population of *H.l.lar* represented by three individuals from previous studies (AF311723, LC548024, and LC548028). These transversion mutations may be due to the prolonged isolation between the postulated southern and northern populations, which are separated by the presence of *H.agilis* in the narrow region between the Perak River and Muda River (Figure 1). Similar findings have been observed in other non-human primates, such as *Macaca* (Rovie-Ryan et al. 2021). Thus, these mutation sites should be used as genetic markers for future taxonomic and genetic assessments involving *lar* gibbons. The southern *H.l.lar* population likely consists of all populations south of the Kangar-Pattani line (Fig. 1) and including the entirety of Peninsular Malaysia. In contrast, the northern population may exist in regions between the Isthmus of Kra and the Kangar-Pattani line. Several previous studies have demonstrated the role of the Isthmus of Kra, the Surat Thani-Krabi depression, and the Kangar-Pattani line as zoogeographical boundaries between Sundaland and Indochina (Baltzer et al. 2007; Woodruff 2010; Hughes et al. 2011; Rovie-Ryan et al. 2013; Abdul-Latiff et al. 2014).

Overall, our findings support the importance of conducting taxonomic and genetic assessments prior to any gibbon translocations and/or reintroductions. The distinguishable differences between the postulated northern and southern *H.l.lar* populations warrant their treatment as separate management units (MUs), a component within the Evolutionary Significant Unit (ESU) (Moritz 1994). Although treated as separate MUs, under the circumstance that the remnant populations of *lar* are showing signs of inbreeding depression or increased fragmentation, mixing (translocation) between MUs are permittable (Moritz 1999).

In summary, we conclude that using the CR segment of the mtDNA, we could taxonomically distinguish *H.l.lar* from the other *H.lar* subspecies, an important result for future translocation and reintroduction programs of rescued and captive gibbons in Peninsular Malaysia. Nevertheless, the use of nuclear DNA data for taxonomic and genetic assessments of captive and rescued gibbons should also be considered, especially for individuals of suspected hybrid origin. Further studies are currently on-going by DWNP (as the authority of wildlife conservation and management) to screen all captive *lar* gibbons in Peninsular Malaysia as well as to collect reference samples from the wild.

## Supplementary materials

Supplementary material 1 Table S1. Data type Specimens data Explanation note Summary table of all used sequences used in this study. https://binary.pensoft.net/file/620462This dataset is made available under the Open Database License (http://opendatacommons.org/licenses/odbl/1.0/). The Open Database License (ODbL) is a license agreement intended to allow users to freely share, modify, and use this Dataset while maintaining this same freedom for others, provided that the original source and author(s) are credited.Millawati Gani, Jeffrine J. Rovie-Ryan, Frankie Thomas Sitam, Noor Azleen Mohd Kulaimi, Chew Cheah Zheng, Aida Nur Atiqah, Nur Maisarah Abd Rahim, Ahmad Azhar Mohammed

Supplementary material 2 Table S2 Data type Variable sites Explanation note Variable regions observed among the Hylobateslar sequences. Blue coloured boxed indicated transversion mutations while green boxes indicated transition mutations. https://binary.pensoft.net/file/620463This dataset is made available under the Open Database License (http://opendatacommons.org/licenses/odbl/1.0/). The Open Database License (ODbL) is a license agreement intended to allow users to freely share, modify, and use this Dataset while maintaining this same freedom for others, provided that the original source and author(s) are credited.Millawati Gani, Jeffrine J. Rovie-Ryan, Frankie Thomas Sitam, Noor Azleen Mohd Kulaimi, Chew Cheah Zheng, Aida Nur Atiqah, Nur Maisarah Abd Rahim, Ahmad Azhar Mohammed

Supplementary material 3 Table S3 Data type Genetic distance Explanation note Pairwise genetic distances among the H.lar sequences used in this study using Kimura-2 parameters (Kimura 1980). https://binary.pensoft.net/file/620464This dataset is made available under the Open Database License (http://opendatacommons.org/licenses/odbl/1.0/). The Open Database License (ODbL) is a license agreement intended to allow users to freely share, modify, and use this Dataset while maintaining this same freedom for others, provided that the original source and author(s) are credited.Millawati Gani, Jeffrine J. Rovie-Ryan, Frankie Thomas Sitam, Noor Azleen Mohd Kulaimi, Chew Cheah Zheng, Aida Nur Atiqah, Nur Maisarah Abd Rahim, Ahmad Azhar Mohammed
